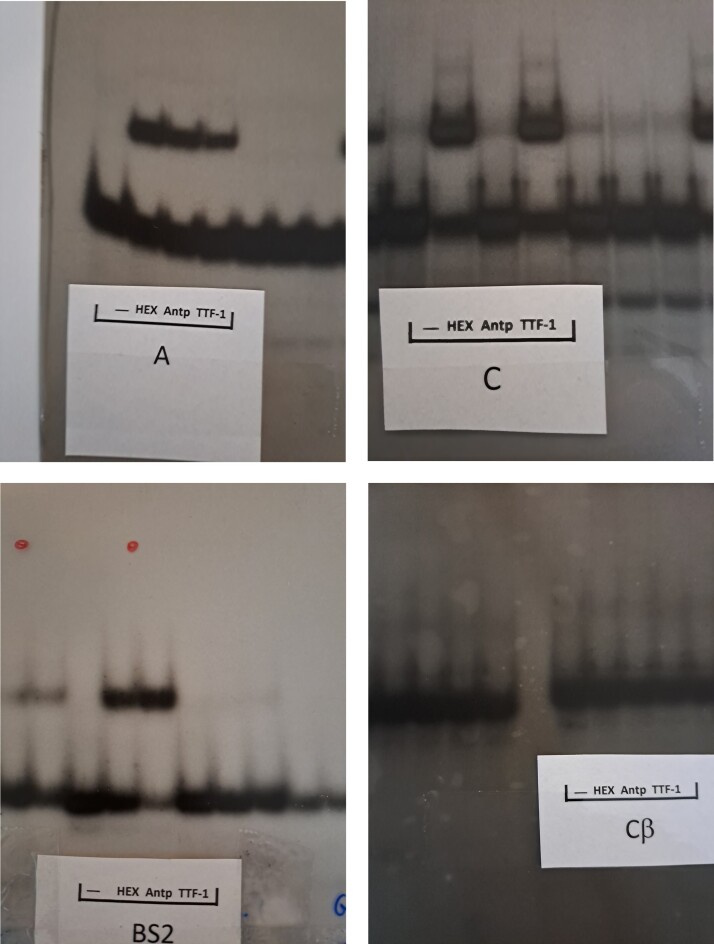# Correction to ‘Expression and function of the homeodomain-containing protein Hex in thyroid cells’

**DOI:** 10.1093/nar/gkad1227

**Published:** 2023-12-18

*Nucleic Acids Research*, Volume 28, Issue 13, 1 July 2000, Pages 2503–2511, https://doi.org/10.1093/nar/28.13.2503

The Editors were alerted in August 2023 that the first lanes (—) of the BS2 and Cβ panels in Figure 6B look very similar.

While investigating the issue, the Editors also found evidence of lane splicing in Figure 6B.

The authors no longer have the original data but provided images of contemporaneous replicate experiments.

These images are provided below along with a new Figure 6B.

The results and conclusion of the article are not affected and remain valid.

New Figure 6B.


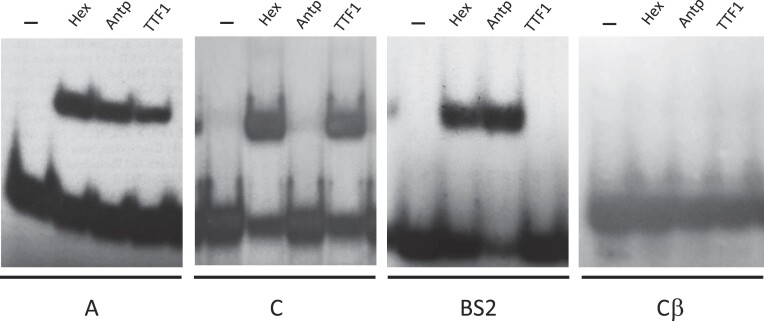


Contemporaneous replicate experiments.